# Direct orientational epitaxy of wafer-scale 2D van der Waals heterostructures of metal dichalcogenides

**DOI:** 10.1093/nsr/nwaf119

**Published:** 2025-03-31

**Authors:** Shenghong Liu, Ke Qin, Jiashu Yang, Tao Hu, Hao Luo, Jingsong Wu, Zhen Cui, Taotao Li, Feng Ding, Xinran Wang, Yuan Li, Tianyou Zhai

**Affiliations:** State Key Laboratory of Materials Processing and Die & Mould Technology, School of Materials Science and Engineering, Huazhong University of Science and Technology, Wuhan 430074, China; School of Materials Science and Engineering, Xi’an University of Technology, Xi’an 710048, China; Suzhou Laboratory, Suzhou 215123, China; State Key Laboratory of Materials Processing and Die & Mould Technology, School of Materials Science and Engineering, Huazhong University of Science and Technology, Wuhan 430074, China; State Key Laboratory of Advanced Technology for Materials Synthesis and Processing, Nanostructure Research Center, Wuhan University of Technology, Wuhan 430070, China; State Key Laboratory of Advanced Technology for Materials Synthesis and Processing, Nanostructure Research Center, Wuhan University of Technology, Wuhan 430070, China; School of Materials Science and Engineering, Xi’an University of Technology, Xi’an 710048, China; School of Integrated Circuits, Nanjing University, Nanjing 210008, China; Suzhou Laboratory, Suzhou 215123, China; School of Integrated Circuits, Nanjing University, Nanjing 210008, China; State Key Laboratory of Materials Processing and Die & Mould Technology, School of Materials Science and Engineering, Huazhong University of Science and Technology, Wuhan 430074, China; Research Institute of Huazhong University of Science and Technology in Shenzhen, Shenzhen 518063, China; State Key Laboratory of Materials Processing and Die & Mould Technology, School of Materials Science and Engineering, Huazhong University of Science and Technology, Wuhan 430074, China; Research Institute of Huazhong University of Science and Technology in Shenzhen, Shenzhen 518063, China

**Keywords:** two-dimensional material, heterostructure, epitaxy growth, optoelectronic device

## Abstract

Two-dimensional (2D) van der Waals (vdW) heterostructures have emerged as a groundbreaking candidate for future integrated circuits due to their tunable band structures, atomically sharp interfaces and seamless compatibility with complementary metal-oxide-semiconductor technologies. Despite their promise, existing synthesis methods, such as mechanical transfer and vapor-phase conversion, struggle to achieve the high-quality, scalable production for practical applications. In response to these longstanding challenges, our study unveils for the first time the direct epitaxial growth of wafer-scale 2D vdW heterostructures (MoS$_2$/SnS$_2$) with exceptional quality and uniformity. This achievement is made possible through fundamentally enhancing the adsorption interactions between intermediates and the underlying material. The heterostructures display pristine, defect-free interfaces, consistent crystal orientation and wafer-level thickness uniformity. The Raman peak shifts of MoS$_2$ and SnS$_2$ are constrained to below 0.5 cm$^{-1}$ across the entire wafer, with intensity deviations maintained within an impressive 2%, and thickness uniformity surpassing 99.5%. Owing to their exceptional crystallinity and interface quality, the heterostructures demonstrate extraordinary electron and hole transfer capabilities, showcasing a prominent rectification effect and an astounding responsivity of $6.28\times 10^3$ A/W, averaged from 30 devices. Our study signifies a pivotal advancement for the integration of 2D materials into semiconductor technologies, paving the way for next-generation integrated circuits.

## Teaser

Epitaxial growth of wafer-scale 2D vdW heterostructures (MoS_2_/SnS_2_) achieved by generating active intermedia, yielding defect-free interfaces, uniform thickness (>99.5%), and exceptional optoelectronic performance.

## INTRODUCTION

The advancement of integrated circuits utilizing silicon semiconductors is increasingly hindered by the physical and practical challenges associated with miniaturizing silicon-based components [1,2]. These constraints have ignited a surge of research into alternative materials for next-generation electronic circuits. Two-dimensional (2D) van der Waals (vdW) heterostructures, composed of stacked layers of diverse 2D materials, provide unparalleled control over electronic and optical properties thanks to their tunable band structures and atomically sharp interfaces. This makes them exceptionally suited for cutting-edge electronic devices [3,4]. Their integration into complementary metal-oxide-semiconductor (CMOS) technologies further amplifies their potential to revolutionize the semiconductor industry by delivering components that boast enhanced efficiency, scalability and versatility, overcoming the limitations of traditional silicon-based technologies [5,6].

Nonetheless, the large-scale synthesis of high-quality 2D vdW heterostructures presents a significant challenge. Current methodologies, including mechanical transfer and sulfurization/selenization, face considerable hurdles [7,8]. While mechanical transfer can yield high-purity isolated flakes, it proves unsuitable for large-scale production due to issues like interfacial impurities, wrinkles and cracks during the transfer process, as well as inconsistent material properties across extensive areas. Such defects compromise the structural integrity and reliability of 2D heterostructures, often limiting their industrial applicability [9,10]. On the other hand, sulfurization and selenization techniques, which enable broader-area growth, are plagued by problems such as lack of control over the orientation and size of crystal domains, random distribution of grain boundaries and possible interlayer contamination. These challenges might prevent the uniformity and scalability critical for commercial applications of 2D heterostructure-based devices.

Considering these formidable challenges, we herein unveil our pioneering achievement for the direct epitaxial growth of wafer-scale 2D vdW heterostructures, characterized by exceptional quality and uniformity. This is accomplished by fundamentally modulating the interactive intermediates to significantly enhance their adsorption on the surface of the first-layer MoS$_2$. The atomic structure of the resulting heterostructures is clearly visible under electron microscopy, displaying distinct vdW interfaces between layers, and exhibiting few intrinsic defects that confirm their high crystallinity. Raman spectroscopy reveals that peak shifts of MoS$_2$ and SnS$_2$ across the wafer do not exceed 0.5 cm$^{-1}$, with intensity variations within $2\%$ of the average, indicating remarkable consistency. Additionally, atomic force microscopy demonstrates a wafer-scale thickness uniformity exceeding $99.5\%$.

Leveraging this high-quality heterostructure material, we fabricated arrayed optoelectronic devices of the 2D vdW heterostructure. The devices exhibit an impressive responsivity of $6.28\times 10^3$ A/W (averaged from 30 devices), with minimal performance variation that seamlessly aligns with statistical norms. Notably, the capability to directly grow such heterostructures at wafer scale signifies a breakthrough in the practical application of 2D materials within semiconductor technologies, providing a viable pathway to surmount the current limitations of silicon-based devices. This direct growth of heterostructures with clean vdW interfaces, alongside the potential for scalable production, positions our approach as a promising solution for next-generation integrated circuits.

## RESULTS AND DISCUSSION

The synthesis of MoS$_2$/SnS$_2$ heterostructures commences with the fabrication of a single-crystal MoS$_2$ wafer, utilizing custom-oriented sapphire substrates aligned with the C-plane and A-plane directions. As shown in the online supplementary material, Fig. S1a illustrates the synthesis process, while Fig. S1b presents the resulting 2-in. single-crystal MoS$_2$ wafer. The wafer displays well-oriented grains, as evidenced by the optical image in Fig. S1c, confirming its suitability for subsequent heterostructure growth.

The characterization of pure MoS$_2$ is provided in Fig. S2, where Raman and second-harmonic generation (SHG) mapping (Fig. S2a and b) validate the crystalline quality and uniformity of the MoS$_2$ wafer. The electron diffraction pattern (Fig. S2c) further corroborates the high crystallinity of the single-crystal MoS$_2$, demonstrating the absence of significant grain boundaries.

**Figure 1.**
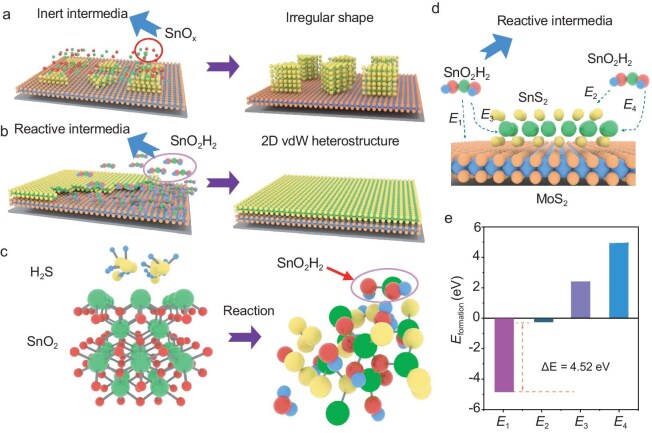
Impact of hydrogen on SnS$_2$ growth on MoS$_2$. (a) Schematic of SnS$_2$ growth on MoS$_2$ without hydrogen, leading to thick SnS$_2$ crystals. (b) With hydrogen, a uniform and thin SnS$_2$ film forms. (c) Hydrogen (H$_2$) aids in breaking Sn-O bonds in SnO$_2$, forming smaller SnO$_2$H$_2$ clusters as precursors for SnS$_2$ thin films. (d) SnO$_2$H$_2$ cluster adsorption at various sites (${E}_1, {E}_2, {E}_3, {E}_4$) with different configurations. (e) Formation energy diagram shows a significant stability difference (${\Delta E}$ = 4.52 eV) for SnO$_2$H$_2$ clusters on MoS$_2$.

We performed polarization-dependent SHG measurements at six points, rotating each from 0$^{\circ }$ to 180$^{\circ }$. Figure S3a shows the SHG signals between 0$^{\circ }$ and 50$^{\circ }$, while Fig. S3b presents the angle-dependent polarization SHG polar plot derived from the extracted signal intensities. According to the test result, the orientation obtained from the fitted polarized SHG signals collected at different points shows consistency, indicating the single-crystalline nature of MoS$_2$ [1,2]. Following the successful synthesis of MoS$_2$, we proceeded with the preparation of the second-layer SnS$_2$. It is first worth noting that direct growth of SnS$_2$ on MoS$_2$ via conventional methods resulted in irregularly shaped flakes distributed unevenly across the MoS$_2$ surface, as shown in Fig. S4. Figure 1 illustrates the impact of environmental conditions on the growth of SnS$_2$ on MoS$_2$ substrates. Employing conventional methods, SnS$_2$ tends to form thick, aggregated crystals, which prevent the development of a continuous and uniform film. This uneven surface morphology introduces grain boundaries and voids, impairing the structural integrity and electrical properties necessary for high-performance optoelectronic devices [11,12]. To overcome these difficulties, we tune the atmosphere to make the reaction media turn from an inert state to an inactive state to enhance the absorption of the media and the surface. In this way, the as-synthesized SnS$_2$ nuclei tends to separate on the surface and the thin and uniform films are easily fabricated. The resulting film exhibits greater smoothness and homogeneity, aligning closely with the material requirements for enhanced electronic and optoelectronic applications. This uniformity in film thickness is critical, as it minimizes defects and improves the mobility of the charge carrier, contributing to superior device performance and stability.

**Figure 2.**
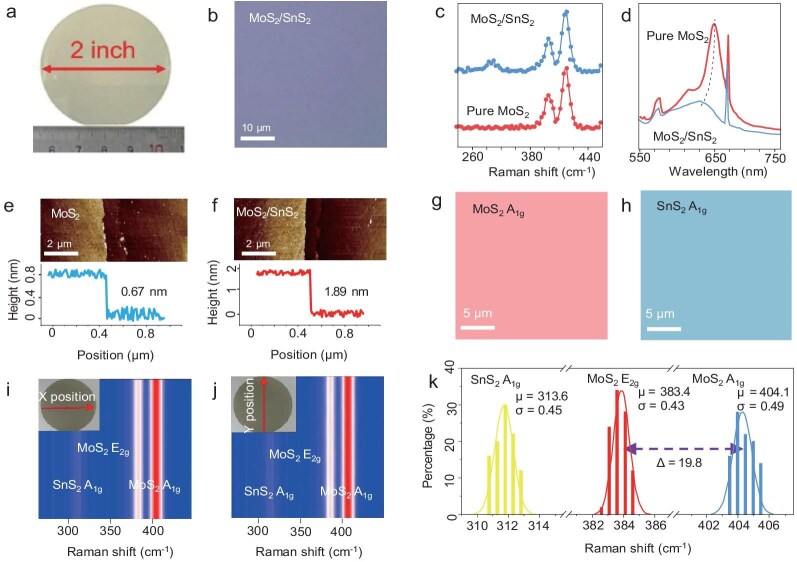
Characterization of a 2-in. wafer of the vdW heterostructure. (a) Photo of a 2-in. heterostructured wafer. (b) Photomicrograph of the heterostructure thin film. (c) Raman comparison chart before and after the two-step CVD process. (d) PL comparison chart before and after the two-step CVD process. (e and f) Thickness comparison charts before and after the two-step CVD process. (g and h) Raman mapping of the thin film at 404 and 314 cm$^{-1}$. (i and j) Pseudo-color Raman map of 55 points in various directions on the 2-in. wafer. (k) Histogram of Raman peak positions.

To further investigate this phenomenon, we conducted *ab initio* molecular dynamics (AIMD) simulations to explore the role of hydrogen in the formation of SnO$_2$H$_2$ clusters. As illustrated in Fig. 1c, hydrogen disrupts Sn-O bonds in SnO$_2$, leading to the formation of smaller SnO$_2$H$_2$ clusters. Comparing to the condition in which hydrogen is absent, the SnO$_2$ precursor is hard to decompose, as the simulated results show in Fig. S5. We also conduct a heating experiment to verify the point. When SnO$_2$ is heated to $600\, {}^{\circ }{\rm C}$ under an inert atmosphere, the rate of weight loss is slower in the absence of H$_2$. However, when 5 sccm of H$_2$ is introduced, the rate of weight loss doubles within 40 minutes (Fig. S6). This experiment demonstrates that the source decomposes more rapidly in an H$_2$ environment. These smaller clusters, generated through hydrogen introduction, are more uniformly distributed on the MoS$_2$ substrate, promoting consistent growth. Figure 1d demonstrates the adsorption of SnO$_2$H$_2$ clusters at various sites on the heterostructure surface (${E}_1, {E}_2, {E}_3$ and ${E}_4$), each with unique stability and formation energies. Here *E*$_1$ refers to the energy associated with the intermediate adsorbed on the SnS$_{2}$ surface, while *E*$_2$ refers to the energy associated with the intermediate adsorbed on the MoS$_{2}$ surface. The formation energy diagram in Fig. 1e reveals a significant energy difference between *E*$_1$ and *E*$_2$ ($\Delta E = 4.52$ eV), indicating a preference for SnO$_2$H$_2$ clusters to adsorb on the MoS$_2$ surface rather than at the edges of the SnS$_2$, thus facilitating uniform SnS$_2$ coverage on the MoS$_2$ substrate [13,14]. We denote by *E*$_3$ and *E*$_4$ the energies associated with the intermediate adsorbed on the SnS$_{2}$ S-zigzag edge and SnS$_{2}$ Sn-zigzag edge, respectively. In our calculated results, both values are positive, indicating that the intermediate clusters are not inclined to adsorb on the edges of SnS$_2$. This suggests that it is difficult to obtain large, aggregated SnS$_2$ structures during the growth process [15]. Using our methodology, we successfully prepared 2-in. wafer-level heterostructure thin films that exhibit excellent color uniformity, as determined by a grayscale histogram analysis (Fig. S7), where more than 95% of the data fall within 1.86 standard deviations ($\sigma$) from the mean (Fig. 2a). Morphological characterization via optical microscopy (Fig. 2b) revealed a uniformly distributed surface without significant fractures or impurities. Raman spectroscopy (Fig. 2c) and photoluminescence (PL, Fig. 2d) tests conducted on samples before and after SnS$_2$ growth demonstrated that the peak positions of the vibrational modes of MoS$_2$ (A$_{\rm 1g}$ and E$_{\rm 2g}$) remained largely unchanged, indicating a relatively weak bond between the two materials. The characteristic peak (A$_{\rm 1g}$) of SnS$_2$ appeared at 313.6 cm$^{-1}$ in the Raman spectrum after SnS$_2$ growth, confirming its successful integration onto the MoS$_2$ film [15,16].

After growth, the PL peak of MoS$_2$ exhibited a significant blue shift from 673 to 653 nm, indicative of a notable electronic transfer between MoS$_2$ and SnS$_2$ [17,18]. We also extracted the full width at half maximum (FWHM) of the peak. Prior to the growth of SnS$_{2}$, the PL FWHM was approximately 28 nm. After growing SnS$_{2}$, the FWHM increased to around 38 nm. The increase in the PL FWHM after SnS$_{2}$ growth can be attributed to the formation of a type-II heterojunction between SnS$_{2}$ and MoS$_{2}$. This leads to a shift in the PL peak and a broader spectral width as a portion of the photogenerated excitons is captured by the energy levels of SnS$_{2}$ (Fig. S8) [18,19]. The atomic force microscope (AFM) test in Fig. 2e and f illustrates the thickness measurements of the grown MoS$_2$ and the subsequent SnS$_2$ layers, revealing approximately three layers of SnS$_2$ atop a single layer of MoS$_2$.

The Raman mapping of MoS$_2$ and SnS$_2$ (Fig. 2g and h) demonstrates uniform distribution over a substantial area, ensuring complete coverage with no discernible noise or artifacts. To assess the uniformity of the heterostructures across the wafer, we analyzed 55 sets of Raman spectra along both the *X* and *Y* axes, respectively, creating pseudocolor maps (Fig. 2i and j) to illustrate variations in Raman peak intensity at each position. Furthermore, we conducted a statistical analysis of the Raman intensity distribution across 110 points on the wafer. The Raman intensity distribution is highly concentrated, which further confirms the uniformity of the prepared heterojunction across the entire wafer (Fig. S9) [1–3]]. Gaussian fitting and histogram analysis of these spectra showed that the standard vibration ($\sigma$) of the peak shifts of MoS$_2$ and SnS$_2$ did not exceed 0.5 cm$^{-1}$, with intensity variations remaining within 2% of the average, thereby confirming excellent consistency across the prepared wafer-level heterostructures (Fig. 2k) [17]. According to the result, the shift difference between the two peaks of MoS$_2$ is 19.8 cm$^{-1}$, which corresponds with the monolayer MoS$_2$ [1].

**Figure 3.**
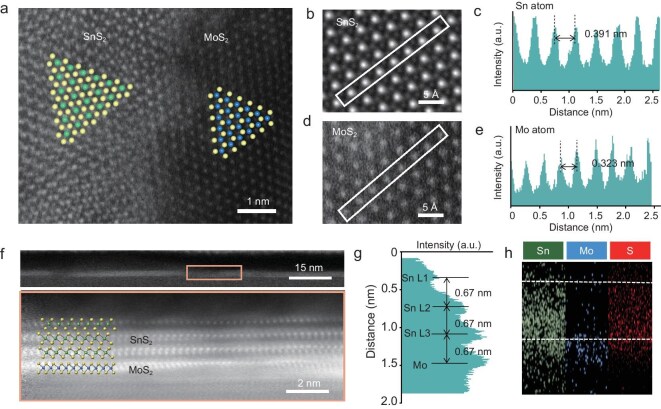
Microscopic characterization of heterostructures. (a) High-resolution STEM image of the MoS$_{2}$/SnS$_{2}$ interface, highlighting atomic arrangements. (b) Enlarged view of the SnS$_{2}$ region. (c) Intensity profile of Sn atoms in SnS$_{2}$, showing 0.391-nm spacing. (d) Enlarged view of the MoS$_{2}$ region. (e) Intensity profile of Mo atoms in MoS$_{2}$, showing 0.323-nm spacing. (f) Cross-sectional STEM image of the MoS$_{2}$/SnS$_{2}$ heterostructure, illustrating atomic stacking with a schematic overlay. (g) Intensity profile across the interface, showing layer spacing and a transition between SnS$_{2}$ and MoS$_{2}$. (h) Elemental mapping of Sn, Mo and S, confirming the composition and uniformity of the layers.

AFM images were also captured from eight distinct regions across the wafer (Fig. S10), demonstrating the flatness of the fully covered heterostructure. The x-ray photoelectron spectroscopy (XPS) presented in Fig. S11 confirms the successful synthesis of the two materials. The binding energy peaks of key elements, including S, Mo and Sn, are clearly identified and match well with the expected reference values. Notably, the peak positions show no significant shifts, indicating the preservation of chemical states and the absence of substantial interactions or distortions in the materials. These results highlight the successful formation of the desired compositions with consistent electronic structures. High-resolution transmission electron microscopy (HRTEM) was employed to unveil the microstructural characteristics of the heterostructures. Initial observations focused on the in-plane characteristics, revealing SnS$_2$ stacking on MoS$_2$ at the sample edges, which had yet to be fully covered. In Fig. 3a, the upper layer is identified as SnS$_2$, while the lower layer is MoS$_2$. Cropped sections of the upper and lower layers (Fig. 3b and d) illustrate interatomic distances between Sn and Mo, measured at 0.391 and 0.323 nm, respectively, aligning with the atomic structural features of the two materials (Fig. 3c and e) [19–21]. Further elemental distribution scans across a larger film area demonstrated uniform distribution of Mo, Sn and S, reinforcing the uniformity of the prepared film (Fig. S12).

Utilizing focused-ion-beam technology, we sectioned portions of the heterostructure samples to examine their longitudinal structural features via electron microscopy. Cuts were made along the sapphire’s A direction (1010) (Fig. S13). As depicted in Fig. 3f, a clear vdW interface between the two materials was observed, with no atomic diffusion or impurities present. Distance measurements between layers indicated a spacing of 0.67 nm between SnS$_2$-SnS$_2$ and SnS$_2$-MoS$_2$ layers, confirming the vdW interactions characteristic of our preparation process (Fig. 3g) [22–25]. Elemental mapping via energy-dispersive x-ray (EDX) spectroscopy clearly revealed a predominance of Mo in the first layer, with Sn predominantly concentrated between the second and fourth layers, while sulfur exhibited a relatively even distribution throughout the heterostructure (Fig. 3h).

During the preparation process, we noted a significant oriented epitaxy for the growth of SnS$_2$ on MoS$_2$. Detailed statistical analysis of the nucleation orientations of SnS$_2$ across various regions of the heterostructure is presented in Fig. S15. The optical microscopy image in Fig. S14 highlights the distribution of SnS$_2$ flakes on MoS$_2$ with varying twist angles. According to the statistical analysis, approximately 52$\%$ of the flakes exhibited a 0$^{\circ }$ twist, while 48$\%$ showed a 60$^{\circ }$ twist, indicative of a bimodal distribution further elucidated by the energy formation diagram (Fig. S15a). The average grain size shown in Fig. S15b is 4.57 $\mu$m.

To explain the statistics of the twist angle, we conduct the theory calculation. Density-functional theory (DFT) calculations were performed to calculate the binding energies ($E_b$) of SnS$_2$ on MoS$_2$ at various rotation angles [26–30]. Figure S16 presents configurations of SnS$_2$ on the MoS$_2$ with different stacking angles and the corresponding binding energies were calculated as $E_b = (E_{\rm total}-E_{\rm MoS_2}-E_{\rm SnS_2})/A$, where $E_{\rm total}$, $E_{\rm MoS_2}$, $E_{\rm SnS_2}$ are the energies of the whole system, MoS$_2$ and SnS$_2$, respectively, and *A* is the area of the supercell of calculation.

MoS$_2$ and SnS$_2$ respectively belong to the P6$\bar{m}$2 and P3$\bar{m}$1 space groups. Considering the crystal symmetry of the single-crystal substrate, the angle between the two materials can be up to 120$^{\circ }$ when forming a heterojunction in the molar superlattice. As shown in Fig. S17a, SnS$_2$ tends to be aligned along two opposite directions, $0^{\circ }$ and $60^{\circ }$, where $0^{\circ }$ corresponds to a local binding energy minimum ($-20.08$ meV/$\mathring{\rm A}^2$) and $60^{\circ }$ corresponds to a global minimum ($-20.21$ meV/$\mathring{\rm A}^2$). These data indicate that SnS$_2$ is more inclined to grow in the 0$^{\circ }$ and 60$^{\circ }$ orientations during epitaxial growth, as schematically illustrated in Fig. S17b, with both orientations being nearly equally probable. In summary, the crystal symmetry of the single-crystal substrate significantly influences the epitaxial growth behavior of 2D materials, demonstrating in particular SnS$_2$’s distinct orientational preference when growing on an MoS$_2$ substrate, with minor differences in binding energy, further confirming its growth advantages in specific directions.

Because of the single-crystal nature of the MoS$_2$ substrate, the growth of SnS$_2$ predominantly aligned along two directions. Electron microscopy revealed consistent orientation characteristics in the lattice fringes across most regions of the SnS$_2$ film (Fig. S18). Notably, a significant lattice mismatch of 14.6% between the two materials resulted in the formation of intriguing Moiré patterns. The electron diffraction pattern in Fig. S19a reveals the stacking relationship between SnS$_2$ and MoS$_2$, with aligned diffraction spots suggesting a $0^{\circ }$ rotational angle, indicative of vdW interaction-driven growth. HRTEM imaging (Fig. S19b) shows lattice fringes within the vdW heterostructure, with a spacing of 2.42 nm, confirming the presence of Moiré fringes due to the stacking configuration consistent with the $0^{\circ }$ alignment of MoS$_2$ and SnS$_2$, as illustrated in Fig. S19c.

We conducted an analysis of the electron diffraction patterns from nine distinct regions of the thin film, revealing nearly uniform orientations across the samples (Fig. S20). Upon magnifying the SnS$_2$ film region, we observed a well-ordered atomic arrangement within the SnS$_2$ crystals, accompanied by a low concentration (about $8.3\times 10^{-11}$ cm$^{-1}$) of sulfur vacancies (Fig. S21). Compared to recent reports, the S vacancies density is relatively low [1,15]. The grain boundary formed by the junction of two opposing SnS$_2$ crystal domains was also observed, which is consistent with the literature (Fig. S22) [3]. Notably, in regions exhibiting step features, the upper SnS$_2$ layers displayed no dislocations during growth and consistently adhered to the AA stacking mode as the number of layers increased (Fig. S23).

**Figure 4.**
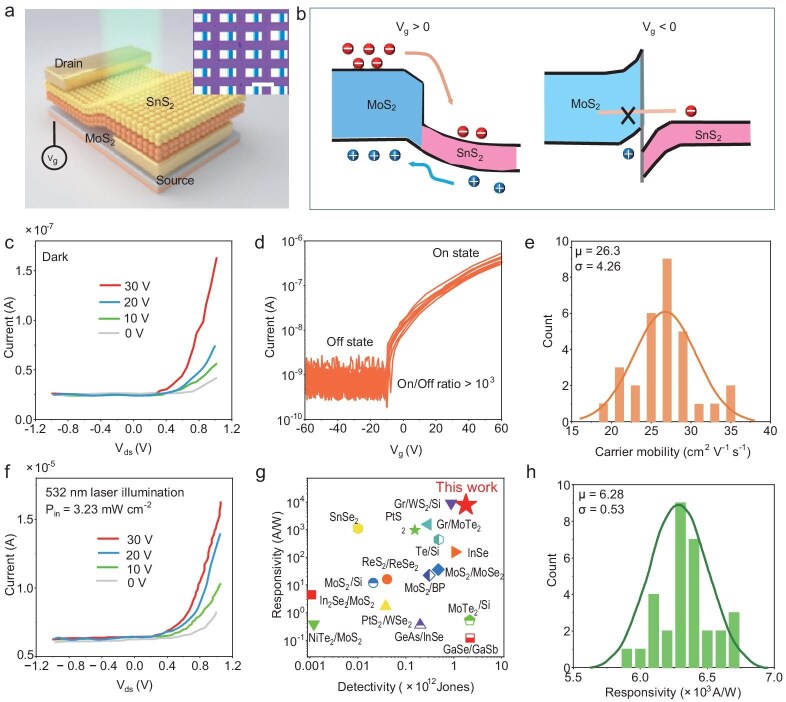
Heterostructure device array performance and characteristics. (a) Schematic of the SnS$_2$/MoS$_2$ heterostructure photodetector under 532-nm laser illumination, with Cr/Au contacts. Inset: optical image of the fabricated array. Scale bar: 100 $\mu$m. (b) Energy band alignment under different gate voltages ($V_{\rm g}$  $>$ 0 and $V_{\rm g}$  $<$ 0). (c) *I*−*V* characteristics at various gate voltages. (d) Transfer characteristic curve ($I_{\rm d}$−$V_{\rm gs}$) showing an on/off ratio $\ge$ 10$^{3}$. (e) Histogram of the carrier mobility with a mean of 26.3 cm$^{2}$/V s. (f) *I*−*V* response under 532-nm illumination. (g) Responsivity versus detectivity comparison of various 2D material devices. (h) Histogram of the responsivity, with a mean of $6.28\times 10^{3}$ A/W.

To assess the potential of these heterostructures in optoelectronic devices, we integrated the samples into array configurations on silicon wafers and systematically evaluated their optoelectronic performance. The device architecture is depicted in Fig. 4a, wherein the top electrode contacts the SnS$_2$ layer, while the bottom electrode interfaces with the MoS$_2$ layer. Detailed fabrication methodologies are provided in the Methods section below. The pronounced band alignment between MoS$_2$ and SnS$_2$ (Fig. 4b) facilitates efficient electron and hole transfer across the heterostructure interface when a positive gate voltage is applied to MoS$_2$. Conversely, applying a negative gate voltage results in a downward shift of the Fermi level in MoS$_2$, impeding charge transfer across the potential barrier formed at the heterojunction.

The *I*−*V* characterization of the devices exhibited a significant rectification effect. As the current transitioned from $-1$ to 1 V, the device current gradually increased from approximately 0 to 17 nA (Fig. 4c). When a positive gate voltage was applied, the current further escalated, reaching 135 nA at 30 V. The calculated rectification factor between $-1$ and 1 V was approximately 300. To evaluate the transistor performance of the device, subsequent testing of the transfer characteristic curves from 30 devices revealed consistent behavior, with current levels rising in response to increasing gate voltages, indicative of n-type modulation characteristics (Fig. 4d). A statistical histogram plotted from the carrier mobility calculated according to the transfer curve demonstrated a normal distribution among the devices. The average carrier mobility, derived from the transfer characteristic curves, was determined to be 26.3 cm$^2$ V$^{-1}$ s$^{-1}$ (Fig. 4e), further underscoring the uniform performance across the device array. Upon illuminating the heterostructure samples with a 532-nm laser at a power density of 3.25 $\mu$W/cm$^2$, we noted a substantial increase in current. At a bias of 1 V, the current reached $1.43 \times 10^{-5}$ A, and with a gate voltage of 30 V, it further increased to $1.67 \times 10^{-5}$ A (Fig. 4f). Responsivity ($R_{\lambda }$) is used to represent the photocurrent generation efficiency, which is defined as $R_{\lambda } = \frac {{I_{\rm photo}}-{I_{\rm dark}}}{P\times S}$, where *P* and *S* are the incident power density and the photodetection area, respectively. The illuminated area (500 $\mu$m$^2$) is as shown in Fig. S24.

Detectivity ($D^{*}$) serves as a parameter to evaluate the sensitivity of a detector, which is defined as $D^{*}=R_{\lambda } \sqrt{S}/\sqrt{(2eI_{\rm dark})}$. The device demonstrated an outstanding responsivity of $7.23 \times 10^3$ A/W and a detectivity of up to $1.76 \times 10^{12}$ Jones (Fig. 4g), highlighting its significant performance advantage compared to other 2D heterostructures [7,31–39] (Fig. 4g). Table S1 within the online supplementary material lists these parameters for different materials. Compared to the MoS$_2$ device without SnS$_2$ growth, there is a significant enhancement in photogain. The comparative data for the MoS$_2$ device are shown in Fig. S25. The responsivity across the 30 devices remained consistent under illumination, indicating strong uniformity and reproducibility (Fig. 4h). Figure S26 illustrates the typical photodetection performance of the heterostructures. The current-time (*I*−*t*) curve shows the response under 532-nm illumination at an incident power density of 1.425 mW/cm$^{2}$, demonstrating periodic fluctuations in the photocurrent. Figure S25b presents the relationship between $R_{\lambda }$ and the photocurrent ($I_{\rm ph}$) as a function of the incident power density ($P_{\rm in}$). The red curve represents the responsivity, which decreases with increasing $P_{\rm in}$, while the blue curve shows the photocurrent, which increases as $P_{\rm in}$ rises. The devices showed excellent reproducibility and strong photo-response uniformity across multiple samples. These results highlight the heterostructure’s potential for high-sensitivity, low-power photodetection, making it a promising candidate for advanced optoelectronic applications.

## CONCLUSION

In summary, this study presents the first successful demonstration of direct epitaxial growth of wafer-scale 2D vdW heterostructures characterized by pristine interfaces, minimal intrinsic defects and exceptional uniformity. This achievement marks a significant advancement in overcoming the limitations of traditional fabrication methods such as mechanical transfer and sulfurization/selenization. The clearly defined atomic structure, featuring distinct vdW interfaces, is complemented by minimal peak shifts (less than 0.5 cm$^{-1}$) across the wafer, underscoring the outstanding consistency of our materials. Additionally, optical microscopy reveals over 95% color uniformity, a critical indicator of film homogeneity. Consequently, the resultant devices exhibit minimal performance variability, achieving a remarkable responsivity of $6.28\times 10^3$ A/W. These results decisively highlight the potential of our approach for scalable production of high-performance devices. Overall, this work represents a pivotal advancement towards the integration of 2D vdW heterostructures in semiconductor technologies, offering a practical and scalable pathway for next-generation integrated circuits that transcend the limitations of conventional silicon-based devices.

## METHODS

### Growth of SnS$_2$ on MoS$_2$ for heterostructure formation

After the successful growth of the MoS$_2$ monolayer, SnS$_2$ was deposited on top of the MoS$_2$ surface. The growth of SnS$_2$ was carried out in the same quartz tube furnace setup used for MoS$_2$ deposition. Tin (IV) oxide (SnO$_2$) and sulfur (S) powders were used as the precursors for the SnS$_2$ growth. The SnO$_2$ powder (99.9% purity, Sigma-Aldrich) was placed in an alumina boat at the center of the furnace, while sulfur was positioned upstream. The pre-grown MoS$_2$ monolayers on sapphire were placed downstream from the SnO$_2$ source. The growth process was optimized by introducing hydrogen (H$_2$) as a reducing agent in addition to argon (Ar) as the carrier gas. The introduction of H$_2$ facilitated the reduction of SnO$_2$ and promoted the formation of smaller SnO$_2$H$_2$ clusters, which are critical precursors for uniform SnS$_2$ film growth. The furnace was heated to $650\, ^{\circ }$C with a constant flow of an Ar/H$_2$ gas mixture (Ar 90%, H$_2$ 10%) at 100 sccm. Sulfur was heated to $180\, ^{\circ }$C, and the resulting vapor was introduced into the furnace to react with SnO$_2$. The reaction produced SnS$_2$, which nucleated on the MoS$_2$ surface. The total growth time for SnS$_2$ was 25 minutes, after which the furnace was naturally cooled to room temperature.

### Characterization

The sample for TEM experiments was fabricated by a dual-focused-ion-beam system (Helios Nanolab G3 UC, FEI). The HRTEM and STEM images were obtained on a double spherical aberration-corrected scanning transmission electron microscope (Titan Themis G2 60-300, FEI) operating at 300 kV. EDS analyses were performed using the Bruker Super-X EDX four-detector system. *I*−*V* characteristics and the pulse measurements were carried out using a probe station (TTPX, Lake Shore) equipped with the semiconductor parameter analyser (B1500A, Keysight) and the semiconductor parameter analyzer (FS-Pro, PRIMARIUS). The Raman spectra and Raman mapping of heterostructures were obtained using confocal Raman spectroscopy (WITec Alpha 300 Raman) with a 532-nm excitation laser at 1 mW. The PL spectra and PL mapping of heterostructures were obtained with a 532-nm excitation laser at 1 mW. XPS (AXIS SUPRA+, Shimadzu) was used to obtain the composition and chemical states.

### DFT calculations

All calculations were performed using projection-augmented wave methods based on DFT [40]. The structural optimization was calculated based on the Perdew-Burke-Ernzerhof package, with convergence criteria for forces and energy of 10$^{2}$ eV Å$^{-1}$ and 10$^{6}$ eV, respectively [41]. The generalized gradient approximation method was used to describe the exchange-correlation function. The DFT-D3 method was used to correct vdW forces, with the cutoff energy set at 550 eV. A vacuum layer of 20 Å was applied and a Brillouin zone of $12\times 12\times 1$ was selected for the K points. The AIMD calculations were performed using the Nosé–Hoover method [42].

### Device fabrication

The bottom electrode (Cr/Au, 10 nm/10 nm) was first fabricated using photolithography and lift-off processes onto silicon wafers with a 300-nm SiO$_2$ dielectric layer. The MoS$_2$/SnS$_2$ heterostructure films were then transferred on the silicon wafers. The top electrodes (Cr/Au, 10 nm/50 nm) were then deposited via electron-beam evaporation, followed by lift-off in acetone.

## Supplementary Material

nwaf119_Supplemental_File
