# Supplementary material for: Direct orientational epitaxy of wafer-scale 2D van der Waals heterostructures of metal dichalcogenides
Source: Natl Sci Rev. 2025 Mar 31;12(5):nwaf119. doi: 10.1093/nsr/nwaf119 (PMC12042757; doi:10.1093/nsr/nwaf119)
Supplement: nwaf119_Supplemental_File [file nwaf119_supplemental_file.pdf]

Supporting Information

Direct Orientational-Epitaxy of Wafer-Scale 2D Van der Waals Heterostructures of Metal Dichalcogenides

Shenghong Liu[1], Ke Qin[2], Jiashu Yang[3], Tao Hu[1], Hao Luo[4], Jinsong Wu[4], Zhen Cui[2,], Feng Ding[3], Taotao Li[5], Xinran Wang[5], Yuan Li[1,6,]*, Tianyou Zhai[1,6,]**

1. State Key Laboratory of Materials Processing and Die & Mould Technology, School of Materials Science and Engineering, Huazhong University of Science and Technology, Wuhan 430074, P. R. China
2. School of Materials Science and Engineering, Xi'an University of Technology, Xi'an 710048, P. R. China
3. Suzhou Laboratory, Suzhou, 215123, P. R. China
4. State Key Laboratory of Advanced Technology for Materials Synthesis and Processing, Nanostructure Research Center, Wuhan University of Technology, Wuhan 430070, P. R. China
5. School of Integrated Circuits, Nanjing University, Nanjing 210008, P. R. China
6. Research Institute of Huazhong University of Science and Technology in Shenzhen, Shenzhen 518063, P.R. China

***Corresponding authors**

Zhen Cui: zcui@xaut.edu.cn

Yuan Li: yuanli1@hust.edu.cn

Tianyou Zhai: zhaity@hust.edu.cn

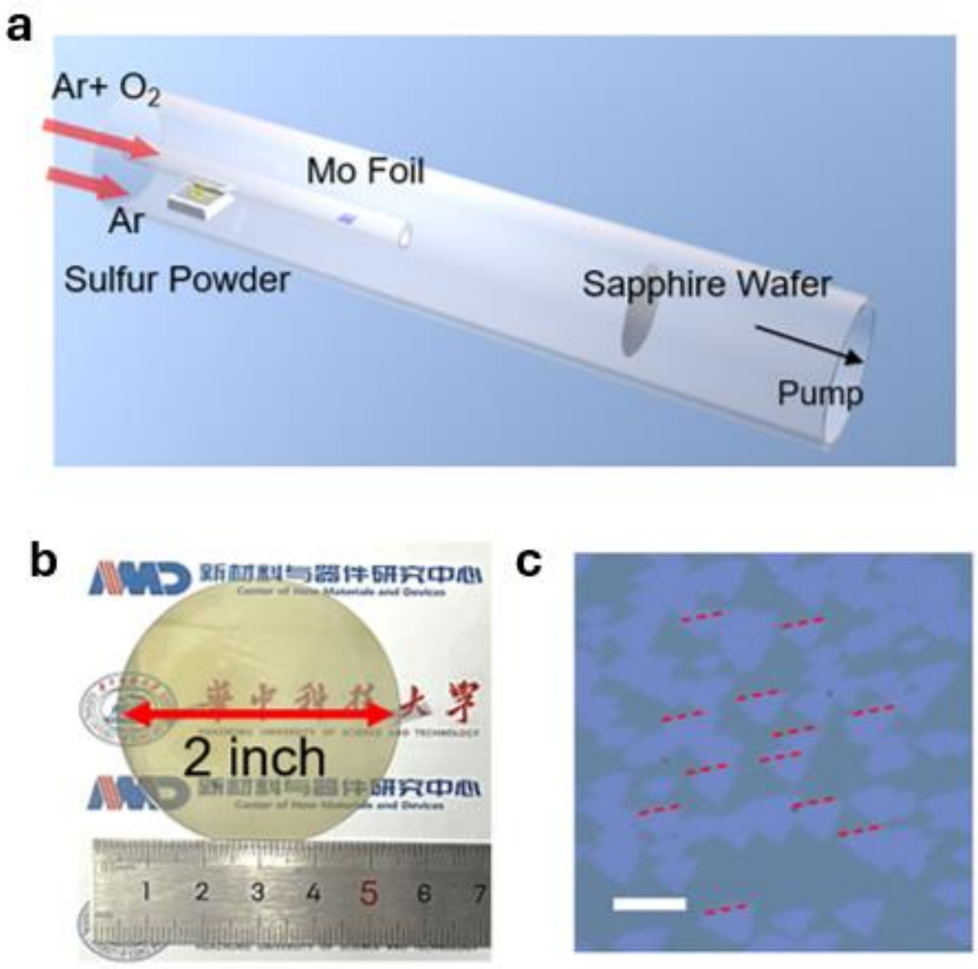

Figure S1. (a) Schematic illustration of the synthesis of the MoS_2 and (b) as-grown single crystal MoS_2 wafer. (c) Optical image of the oriented grains of MoS_2. Scale bar: 3 μm.

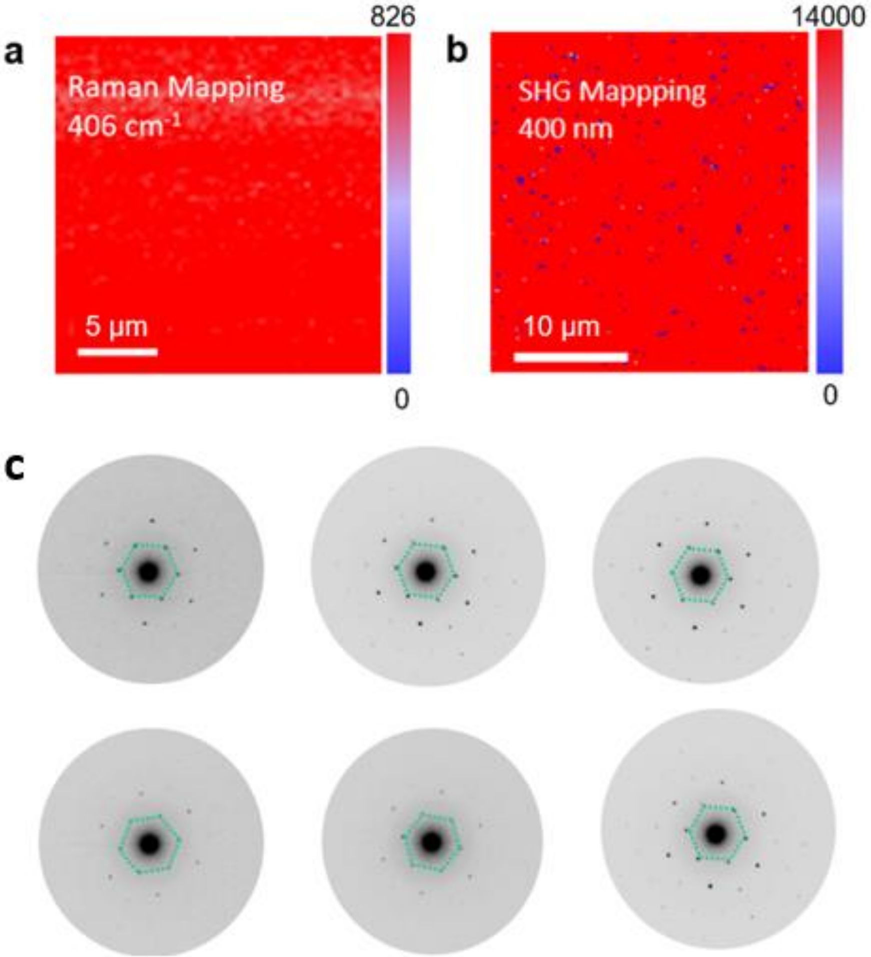

Figure S2. (a, b) Raman and second harmonic generation mapping of the pure MoS_2.

(c) The electron diffraction of the single crystal MoS_2.

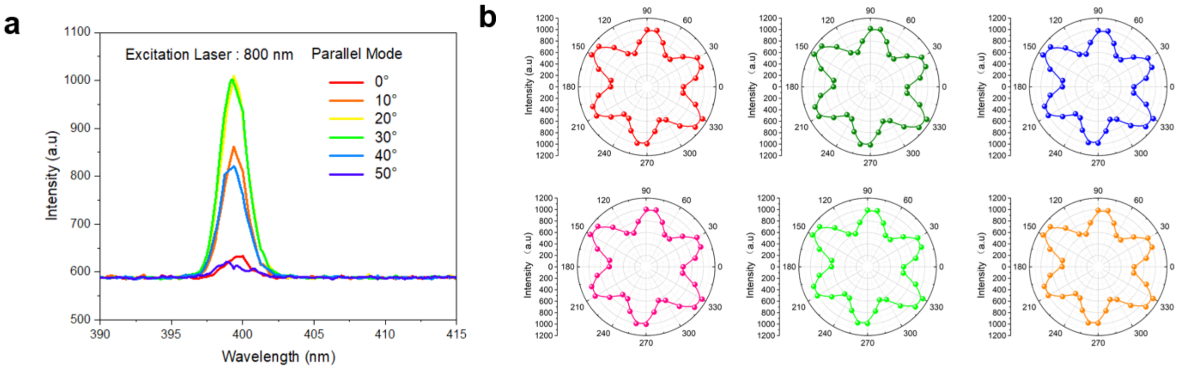

Figure S3. (a) The SHG signal of the sample at different rotation angles. (b) Polarized SHG of the MoS_2 at 6 different points.

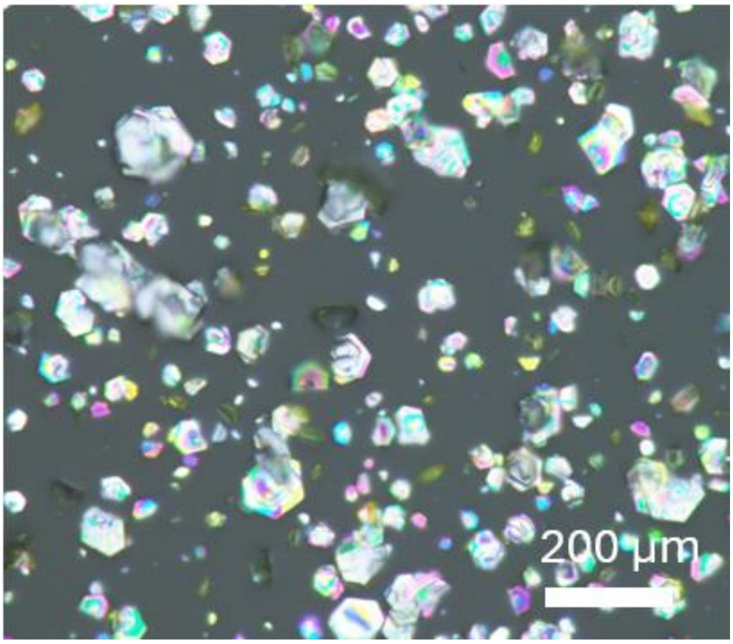

Figure S4. Optical image of irregularly shaped white SnS_2 crystals distributed on the surface of MoS_2.

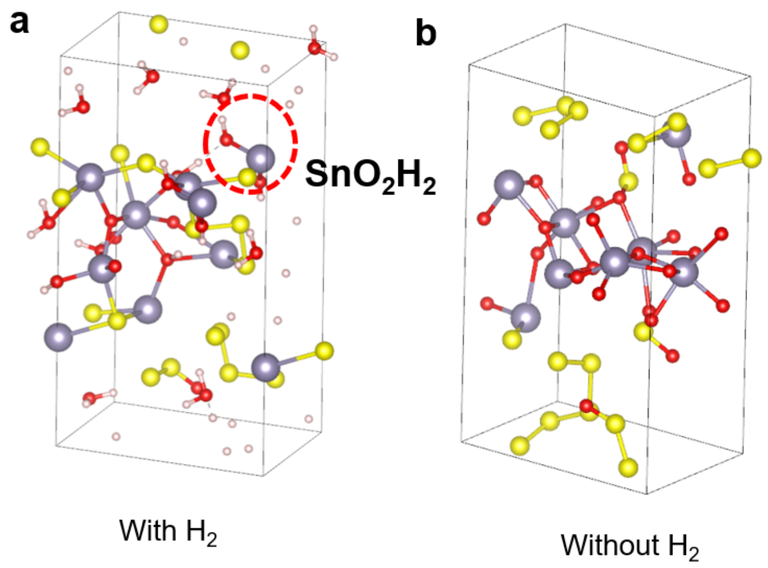

Figure S5. The MD simulation result comparison.

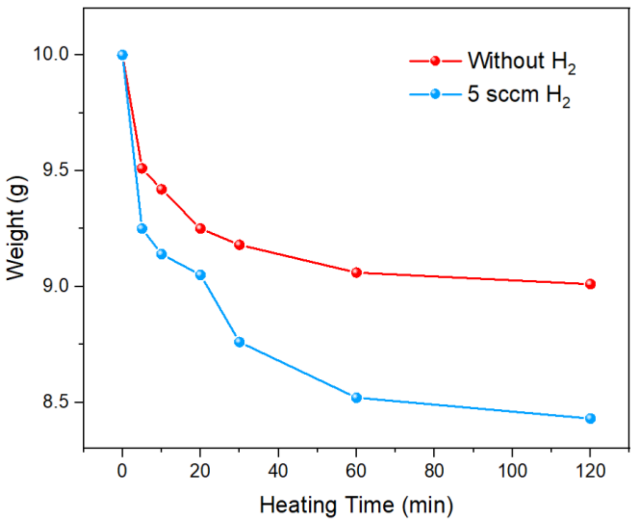

Figure S6. Weight loss of SnO2 under heating conditions.

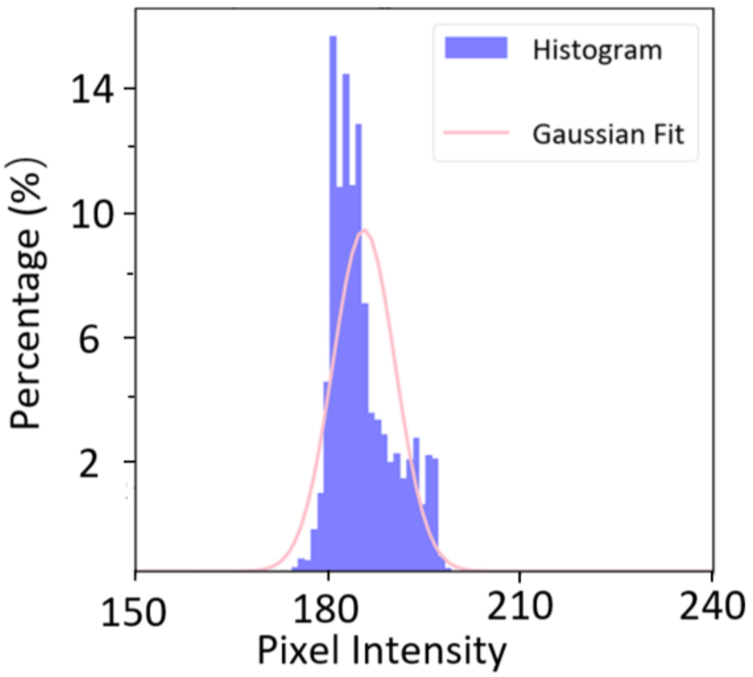

Figure S7. Histogram of pixel intensity distribution showing a Gaussian fit.

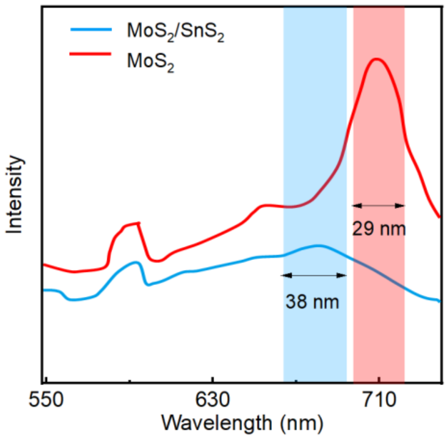

Figure S8. Measured Full Width at Half Maximum (FWHM) of the MoS_2 Photoluminescence (PL) Spectrum.

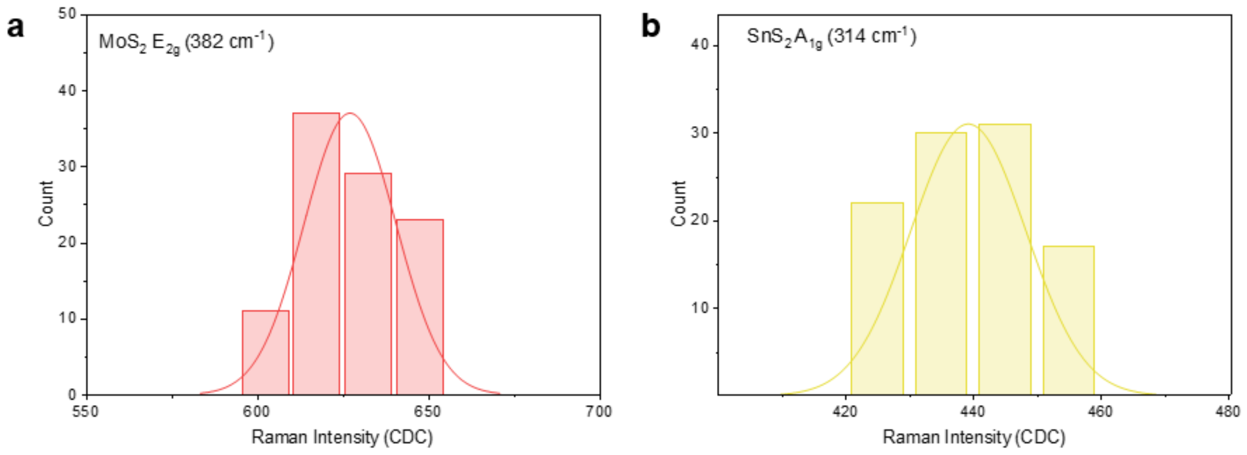

Figure S9. Statistical information of Raman intensity at different points.

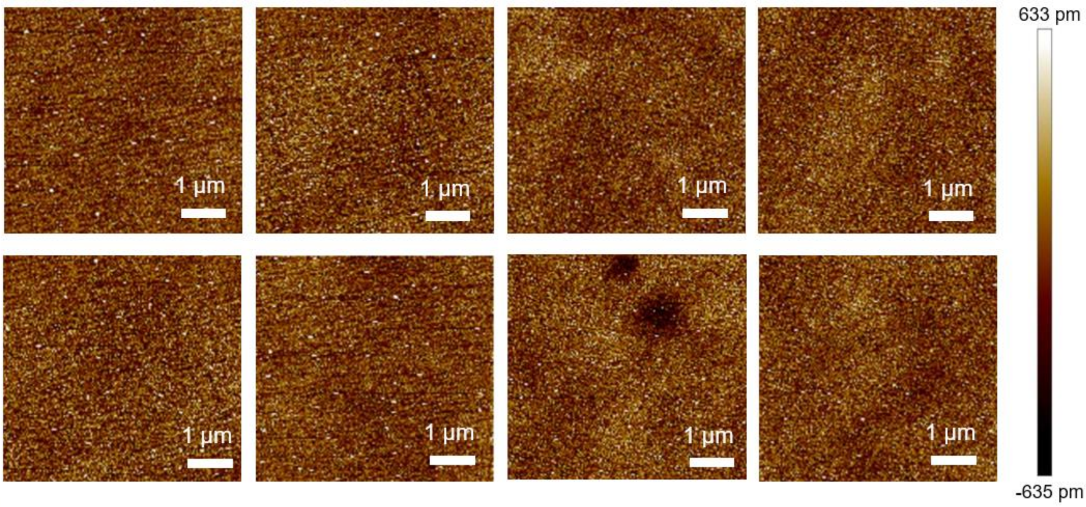

Figure S10. The AFM image of the Heterostructure are 8 different points.

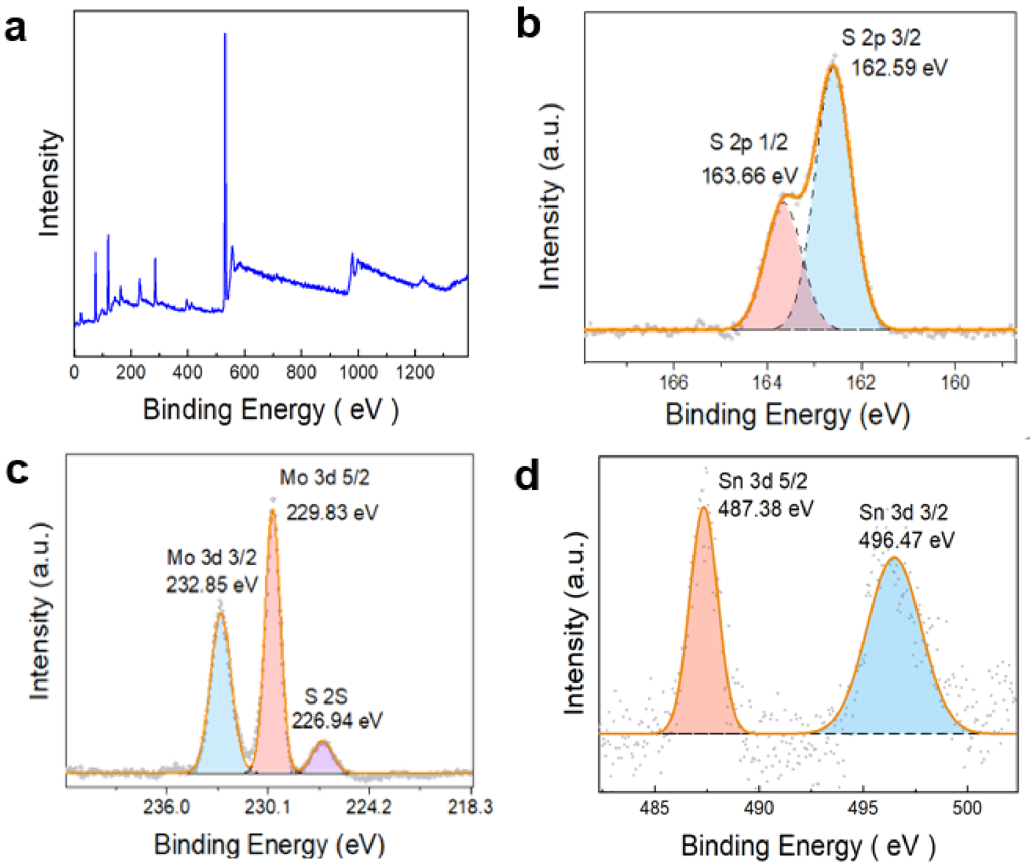

Figure S11. (a) Survey scan of the MoS_2/SnS_2 heterostructures. (b-d) High-resolution XPS spectra and the corresponding curves of S 2p, Mo 3d and Sn 3d, of the MoS_2/SnS_2 heterostructures.

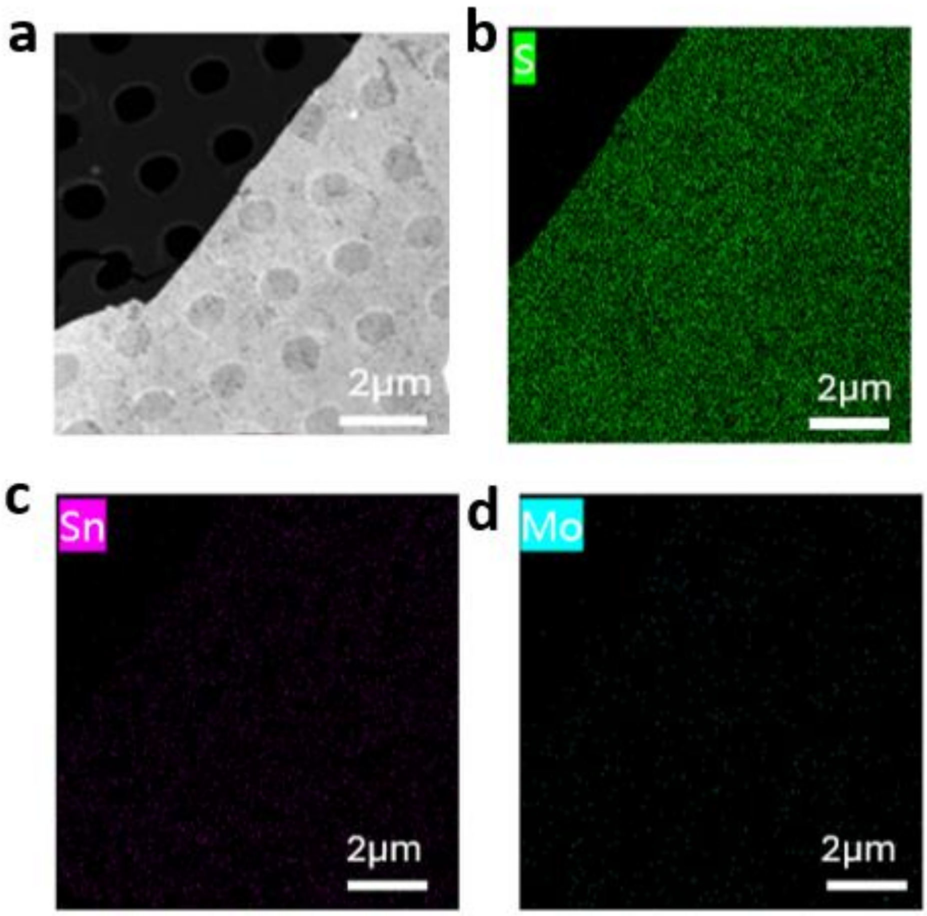

Figure S12. EDX mapping of the film (a) sample on the Cu grid and (b-d) relative element distribution including S, Sn and Mo.

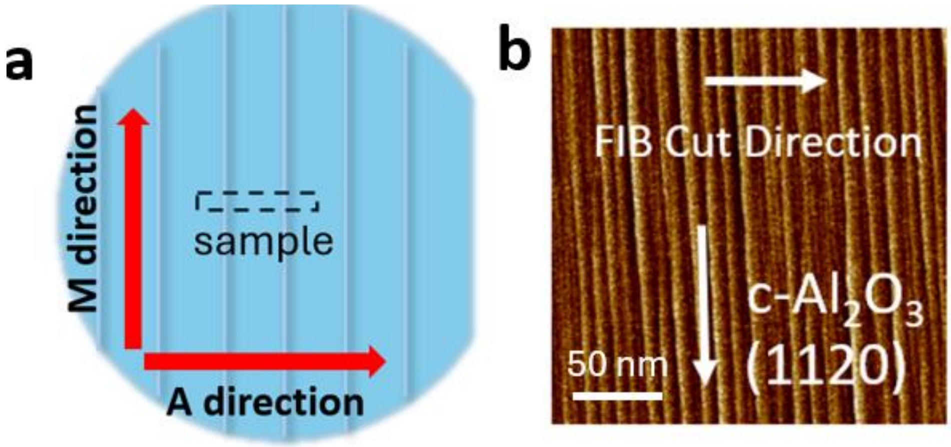

Figure S13. (a) Schematic illustration of the cut-sample, (b) AFM image of the oriented step on the sapphire surface.

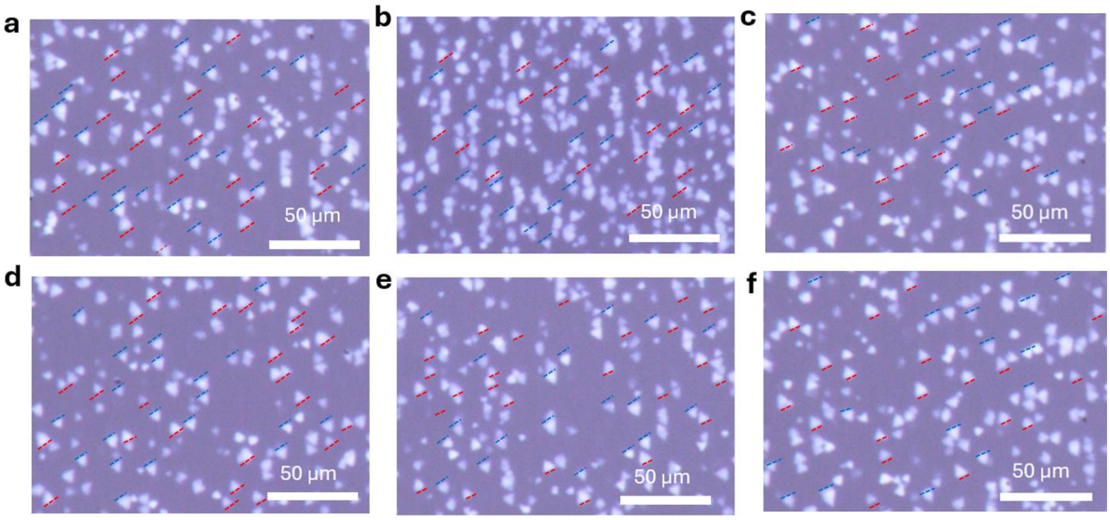

Figure S14. Statistical Analysis of SnS_2 nucleation orientations in different regions.

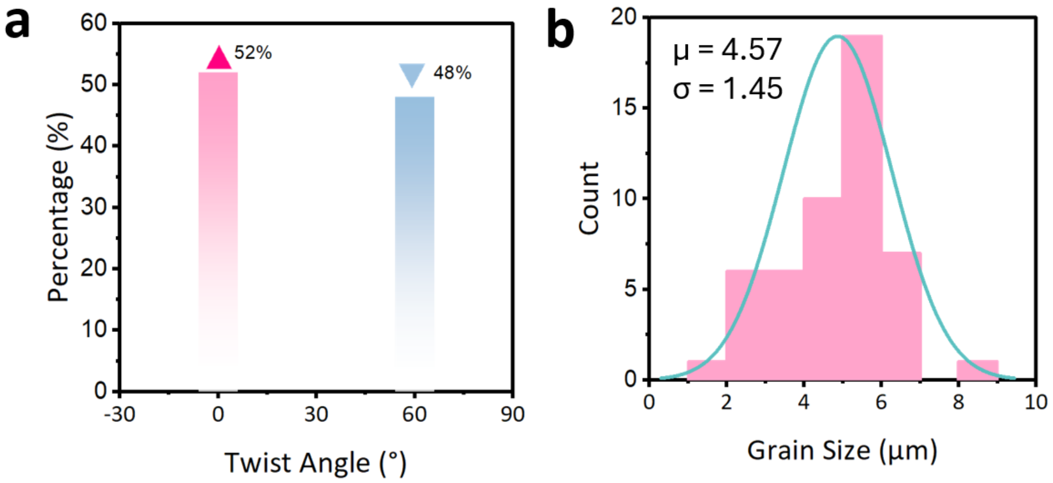

Figure S15. (a) Twist angle distribution of SnS_2/MoS_2 heterostructures, with ~52% at 0° and ~48% at 60°. (b) Grain size distribution histogram.

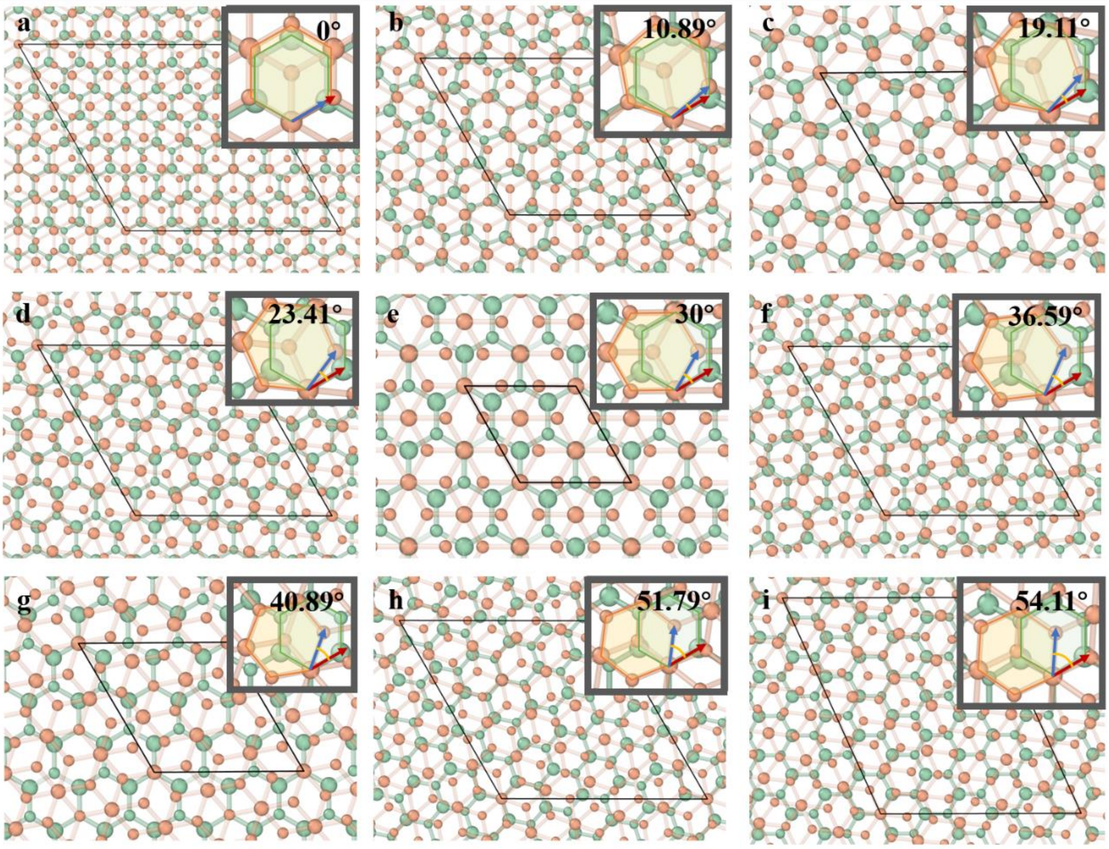

Figure S16. Schematic diagram of MoS_2-SnS_2 stacking at different angles. The stacking details and interlayer angle of MoS_2 (green) and SnS_2 (orange) layers are illustrated in the top-right corner, with the black border delineating the primitive cell of the stacked structure.

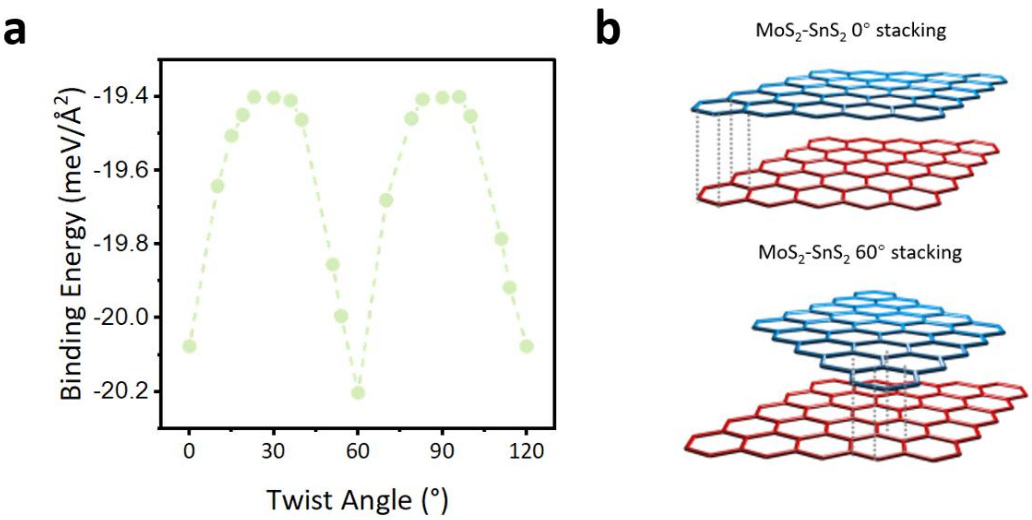

Figure S17. (a) The binding energy of MoS_2-SnS_2 heterostructures under various stacking twist angles demonstrates significant advantages at 0° and 60° stacking configurations. (b) Schematics of 0-degree and 60-degree SnS_2 on MoS_2 stacking.

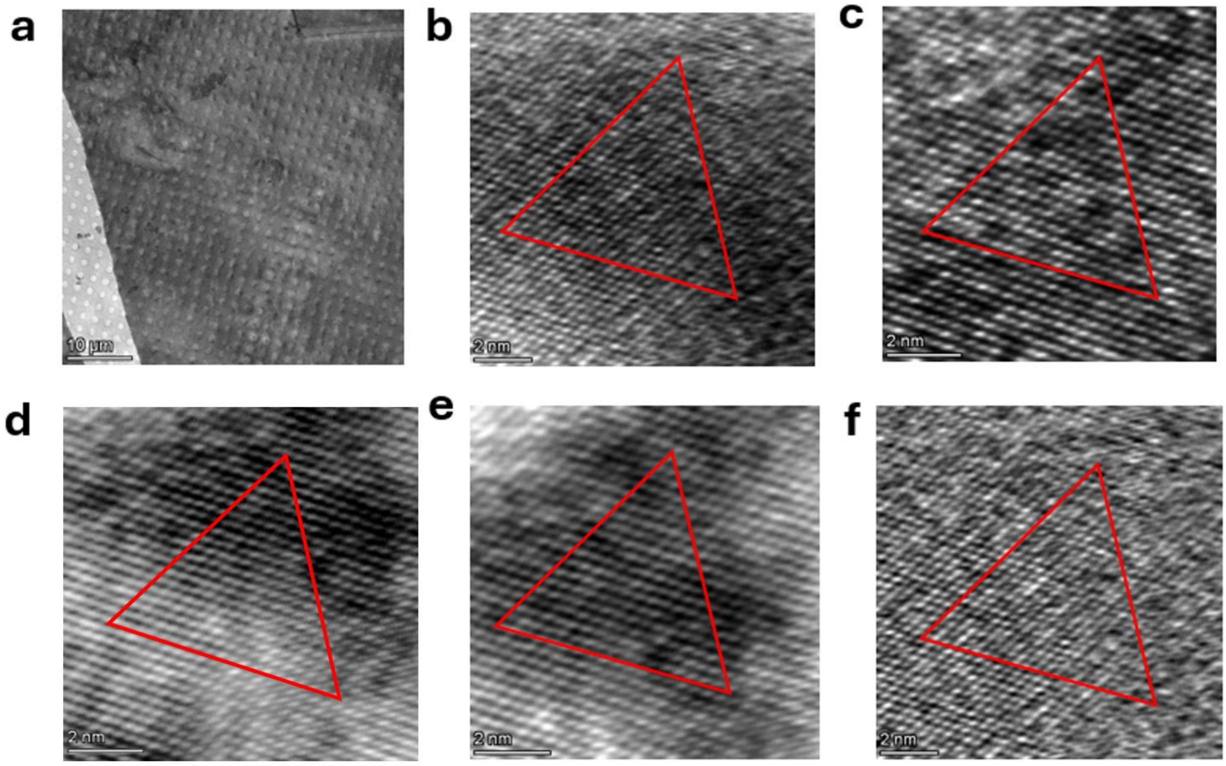

Figure S18. Lattice fringe orientation of SnS_2 on MoS_2.

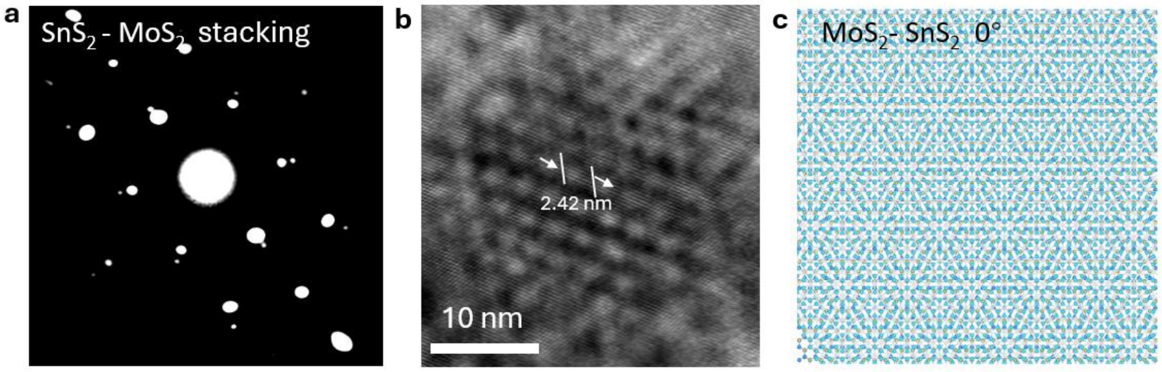

Figure S19. (a) Electron diffraction pattern showing the stacking relationship between SnS_2 and MoS_2. The diffraction spots indicate a 0-degree rotation angle between SnS_2 and MoS_2, suggesting an orientation growth relationship induced by van der Waals forces. (b) High-resolution transmission electron microscopy (HRTEM) image displaying the lattice fringes of SnS_2. The spacing of 2.42 nm indicates the Moiré fringes forms. (c) Schematic model illustrating the 0-degree stacking alignment between MoS_2 and SnS_2.

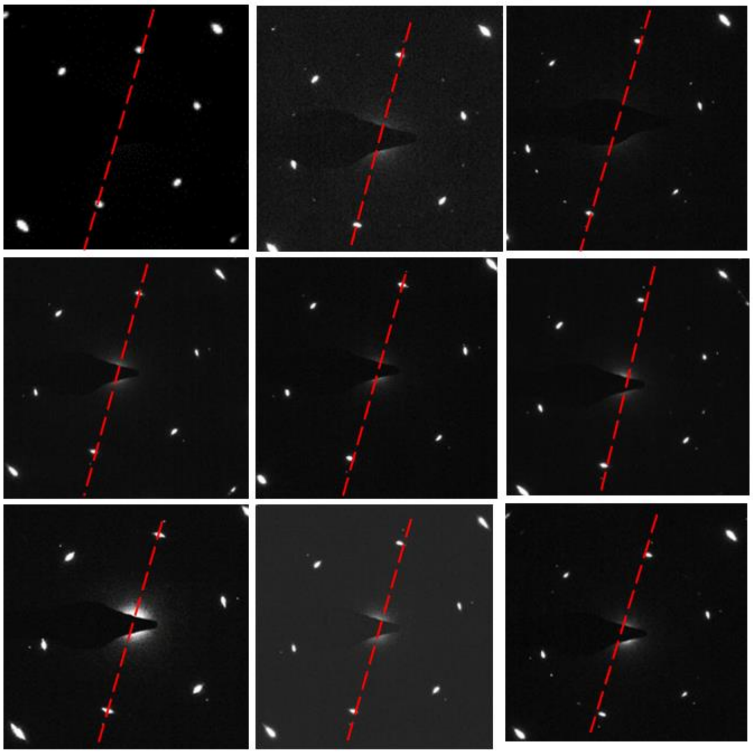

Figure S20. Electron diffraction shows consistent orientation at different Locations.

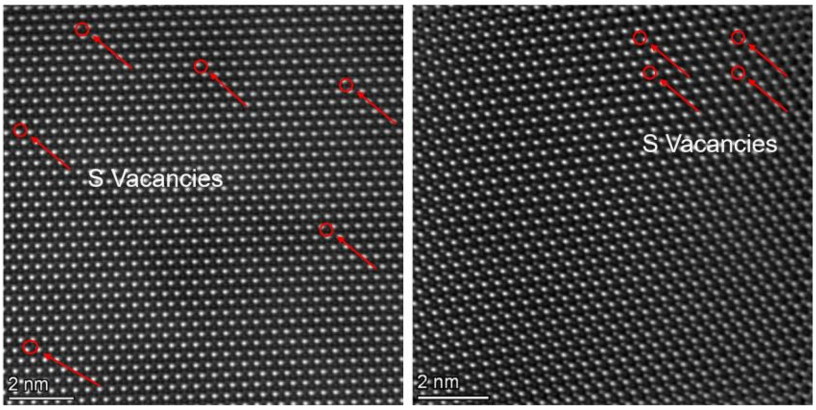

Figure S21. Atomic structure of the SnS_2 surface with low S vacancies density

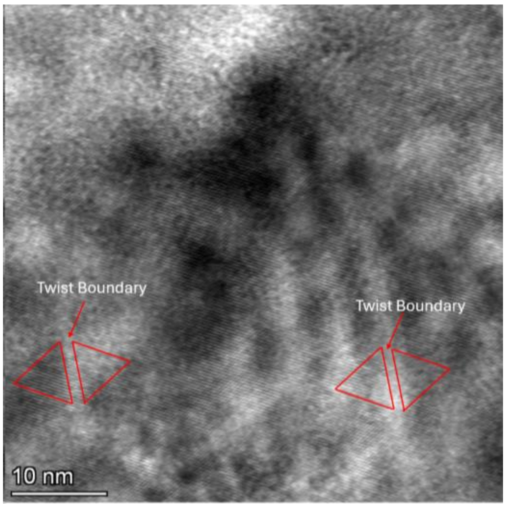

Figure S22. The TEM image of SnS_2 with twist boundaries annotated above.

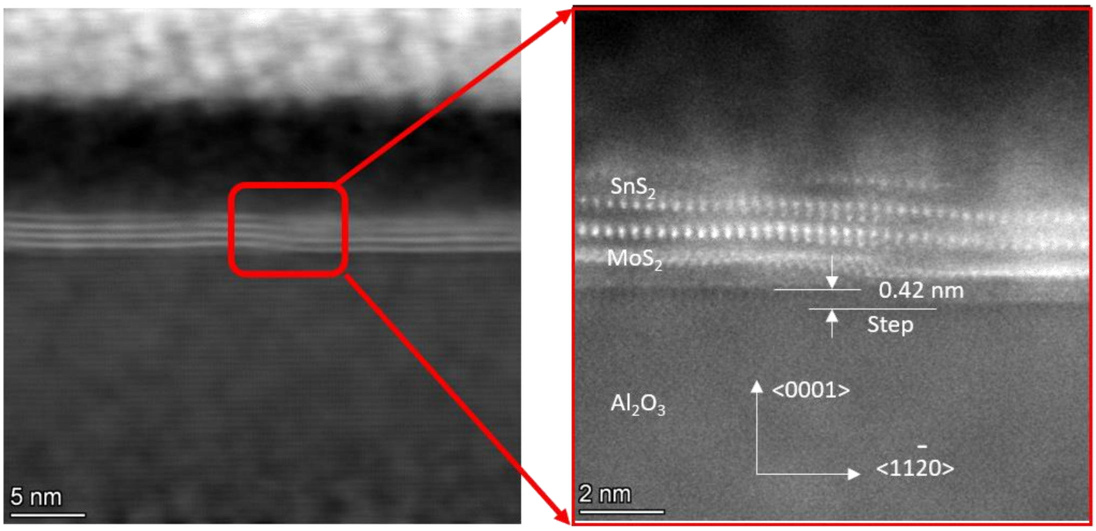

Figure S23. HR-TEM of the Heterojunction cross-section at the sapphire step edge.

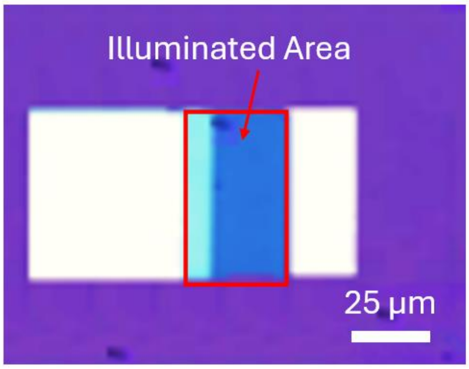

Figure S24. Optical image of the heterostructure device and the box area are specified the illuminated area.

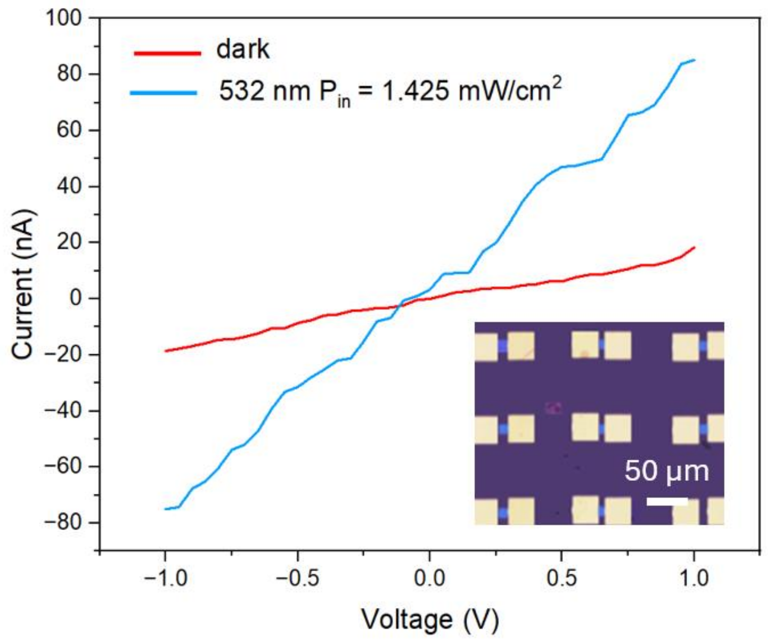

Figure S25. The current-voltage (I-V) characteristics of a device under two conditions: in the dark (red) and under 532 nm light. Inset: optical image of the MoS_2 device.

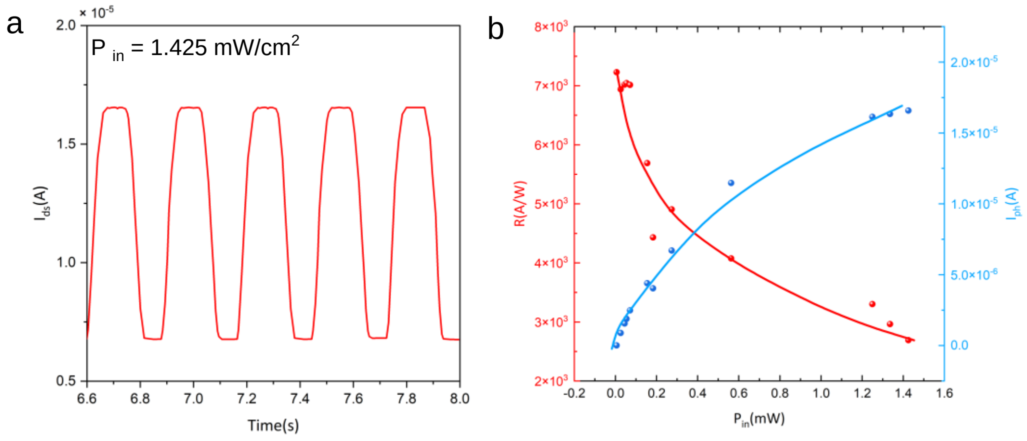

Figure S26. The photodetection performance of the heterostructures. (a) I-T curve under 532 nm illumination and (b) the Responsivity (R) and photocurrent (I_{ph}) varied with the incident power density (P_{in}).

Table S1. The Comparative Parameters of Different Materials.

Materials	Responsivity (A/W)	Detectivity (Jones)	Channel Dimension
In_2Se_3/MoS_2	4.47	1.07×10^{9}	L: 15 μm W: 20 μm
$ReS_2/ReSe_2$	16.24	4.06×10^{10}	L: 25 μm W: 22 μm
PtS_2/WSe_2	1.7	3.80×10^{10}	L: 10 μm W: 20 μm
$NiTe_2/MoS_2$	0.39	1.20×10^{9}	L: 40 μm W:·20 μm
$MoS_2/MoSe_2$	36	4.80×10^{11}	L: 40 μm W:·60 μm
MoS_2/BP	22.3	3.10×10^{11}	L: 30 μm W:·16 μm
GeAs/InSe	0.36	2.00×10^{11}	L: 20 μm W: 10 μm
GaSe/GaSb	0.12	2.20×10^{12}	L: 26 μm W: 18 μm
SnS_2/MoS_2	28	4.00×10^{11}	L: 17 μm W: 15 μm
$MoTe_2$/Si	0.526	2.17×10^{12}	L: 20 μm W: 27 μm
MoS_2/Si	11.9	2.10×10^{10}	L: 20 μm W: 30 μm
Te/Si	437.24	4.86×10^{11}	L: 17 μm W: 30 μm
Gr/WS_2/Si	8960	8.86×10^{11}	L: 10 μm W: 40 μm
PtS_2	1560	2.90×10^{11}	L: 18 μm W: 20 μm
InSe	157	1.07×10^{12}	L: 30 μm W: 16 μm
$SnSe_2$	1100	1.01×10^{10}	L: 18 μm W: 20 μm
Gr/$MoTe_2$	970	1.55×10^{11}	L: 20 μm W: 40 μm
This Work	7230	1.76×10^{12}	L: 10 μmW: 50 μm
